# Meconium microbiota in naturally delivered canine puppies

**DOI:** 10.1186/s12917-024-04225-2

**Published:** 2024-08-12

**Authors:** Alessia Bertero, Penelope Banchi, Angela Del Carro, Michela Corrò, Barbara Colitti, Ann Van Soom, Luigi Bertolotti, Ada Rota

**Affiliations:** 1 Department of Veterinary Sciences, University of Turin, 10095 Grugliasco (TO), Italy; 2 Department of Internal Medicine, Reproduction and Population Medicine, Faculty of Veterinary Medicine, Ghent University, 9820 Merelbeke, Belgium; 3 Istituto Zooprofilattico Sperimentale delle Venezie, 35020 Legnaro (Padua), Italy

**Keywords:** Dog, Puppy, Meconium, Bacteria, Culture, Next-generation sequencing

## Abstract

**Background:**

Microbial colonization during early life has a pivotal impact on the host health, shaping immune and metabolic functions, but little is known about timing and features of this process in dogs. The objectives of this study were to characterize the first step of intestinal microbiota development in naturally delivered canine puppies and to investigate its relationship with the maternal bacterial flora, using traditional culture and molecular analyses. Sixty puppies of two breeds, Appenzeller Cattle Dog (*n* = 3 dams) and Lagotto Romagnolo (*n* = 6), housed in the same breeding kennel, were included in the study. Swabs were collected in duplicate (for culture and for molecular analysis) from the dams’ vagina and rectum at the end of parturition, from puppies’ rectum, before maternal care, and from the environment (floor of the nursery and parturition box).

**Results:**

93.3% meconium samples showed bacterial growth, limited to a few colonies in 57.0% of cases. High growth was detected for *Enterococcus faecalis*, which was the most frequently isolated bacterium. The genus *Enterococcus* was one of the most represented in the dams’ rectum and vagina (88.9% and 55.6%, respectively). The genera *Staphylococcus*, *Enterococcus*, *Escherichia* and *Proteus* were also often isolated in meconium but were usually present in maternal samples as well, together with ubiquitous bacteria (*Acinetobacter*, *Psychrobacter*). In the environmental samples, just a few bacterial species were found, all with low microbial load. Additionally, bacteria of the phyla Proteobacteria, Firmicutes, and Actinobacteria were identified in meconium through molecular analysis, confirming the culture results and the early colonization of the newborn gut. Maternal, meconium and environmental samples had similar alpha diversity, while beta-diversity showed differences among families (i.e. a dam and her litter), and association indexes revealed a significant correlation between family members and between sample origin, suggesting a strong contribution of the maternal flora to the initial seeding of the canine neonatal gut and a strong individual dam imprint.

**Conclusion:**

This study showed that the meconium of vaginally delivered puppies has its own microbiota immediately after birth, and that it is shaped by the dam, which gives a specific imprint to her litter.

**Supplementary Information:**

The online version contains supplementary material available at 10.1186/s12917-024-04225-2.

## Background

Microbial colonization during early life represents a key developmental process that shapes the host immunity and metabolism [1]. Most microbial communities live symbiotically in the intestine, and the establishment of the gut microbiome is fundamental for the development of the neonatal immune system, for defence against enteric infections and for future health. The disruption of the microbial communities has been linked to the occurrence of various chronic diseases in humans [2] and in dogs [3].

The possibility to optimize or modulate the microbial ecosystem relies on understanding its origin and knowing the timing of initial seeding; these aspects have been the object of extensive research in humans and in animals. Traditionally, the human microbiota is thought to be acquired during and after birth, but the ‘sterile womb’ paradigm (i.e., the biological dogma that a human foetus is sterile), has been challenged, and the possibility of in utero colonization has been investigated with molecular approaches [4, 5]. Studies on dogs led to the isolation of bacteria from foetuses and placentae [6, 7], but a more recent investigation accounting for the high risk of contamination showed that the bacteria load in canine pregnancies is very low and likely of environmental origin [8].

Meconium is formed before birth, is rapidly colonized by bacteria in human neonates [9], and the bacterial load and diversity increase over time after birth [2]. The more direct contact with the environment, together with differences in maternal care and the presence of littermates might impact the initial microbial colonization in puppies compared to other species. Knowledge of the microbial flora of meconium and of its colonization timing and pathways in newborn puppies is limited [7, 10].

The objectives of this study were to characterize the first step of intestinal microbiota development in naturally delivered canine puppies and to investigate its relationship with the maternal bacterial flora, using traditional culture and molecular analyses.

## Results

The nine litters included in the study amounted to sixty puppies, twenty-one Appenzeller Cattle Dog (ACD) and thirty-nine Lagotto Romagnolo (LR). The mean birth weight was considered normal by the breeder for the dams’ lineage [(g) ± SD: ACD 406 ± 48; LR 251 ± 76]. All the stillbirths (Table 1) were due to prolonged parturition, as suggested by the monitoring of the birthing progress and the immediate examination of the newborns.

**Table 1:** Purebred litters included in the study and breed, age, weight, and parity of their dams

Litter	Breed	Puppies (*N*)		Dam ID	Age (years)	Weight (Kg)	Parity (*N*)
		Alive	Stillborn				
1	ACD	8	0	A01	6.5	25.6	4
2	ACD	6	3	A02^1^	4.2	22.8	3
3	ACD	4	0	A05^1^	6.0	26.4	4
				**Mean ± SD**	**5.6 ± 1.2**	**24.9 ± 1.9**	**3.7 ± 0.6**
4	LR	8	1	A04^1^	5.6	12.5	5
5	LR	7	1	A07	1.5	13.6	1
6	LR	3	1	A08	7.6	12.4	6
7	LR	4	0	A09^1^	3.2	13.8	2
8	LR	6	0	A03	1.5	12.4	1
9	LR	8	0	A06	3.9	14.2	4
				**Mean ± SD**	**3.9 ± 2.4**	**13.2 ± 0.8**	**3.2 ± 2.1**

ACD = Appenzeller Cattle Dog; LR = Lagotto Romagnolo. Mean values ± standard deviation (SD) in bold

^1^Animals used in NGS analyses

### Culture

Only four meconium samples from two litters resulted negative in culture (6.7%) whereas 93.3% of the samples was positive for bacterial growth. The number of bacterial species detected in a single sample varied from none to six. Both Gram-positive and Gram-negative bacteria were identified and are presented in the Supplementary Material along with their frequency of isolation.

Growth in culture was limited to few colonies in 57.0% of cases, high growth was detected rather frequently (38.9% cases), especially for *Enterococcus faecalis*, while moderate growth was detected in 4.1% of samples.

The number of bacterial species (*n* = 17) isolated from the nine vaginal swabs (V) was higher compared to that isolated from the nine rectal swabs (R) of the bitches (*n* = 13). Forty-eight times a match between puppies and their dam was observed for at least one bacterial species. Specifically, a match with vaginal bacteria was observed 43 times in meconium samples and 28 times between the rectal flora of the dam and the meconium. The bacterial species isolated from the meconium and from the rectal and vaginal swabs of the dams are listed in Table 2 and detailed in the supplementary material (S1).

**Table 2:** Bacterial species identified in the samples, divided for sample origin

	Gram-positive bacteria		
	Meconium	Vagina	Rectum
***Staphylococcus***	X	X	X
*S. equorum*	X		
*S. haemolyticus*	X		
*S. lentus*	X		
*S. napalensis*	X		
*S. saprophyticus*	X	X	
*S. sciuri*	X	X	
*S. simulans*	X		X
*S. xylosus*	X		
*S. aureus*	X	X	
*S. pseudintermedius*	X	X	
***Streptococcus***		X	
*S. canis*		X	
***Enterococcus***	X	X	X
*E. canintestini*	X	X	X
*E. canis*			X
*E. faecalis*	X	X	X
*E faecium*	X		
*E. hirae*			X
***Macrococcus***	X	X	
*M. canis*	X		
*M. caseolyticus*	X	X	
***Lactobacillus***			X
*L. murinus*			X
***Micrococcus***	X		
*M. luteus*	X		
***Clostridium***	X	X	X
*C. perfringens*	X	X	X
***Bacillus***	X	X	
*B. cereus*		X	
*B. pumilus*	X		
***Aerococcus***	X		
*A. viridans*	X		
***Kurthia***		X	
*K. zopfii*		X	
***Escherichia***	X	X	X
*E. coli*	X	X	X
*E. coli (haemolytic)*		X	X
***Klebsiella***	X	X	X
*K. oxytoca*	X		
*K. pneumoniae*	X	X	
*K. variicola*			X
***Proteus***	X	X	X
*P. mirabilis*	X	X	X
***Leclercia***	X		
*L. adecarboxylata*	X		
***Acinetobacter***	X		
*A. lwofii*	X		
*A. radioresistens*	X		
***Enterobacter***	X		
*E. cloacae*	X		
***Citrobacter***			X
*C. freundii*			X
***Psychrobacter***	X	X	X
*P. pasteurii*	X		
*P. sanguinis*	X	X	X
***Pantoea***	X		
***Glutamicibacter***	X		

X = positive sample

Some bacterial species were exclusively isolated from the meconium. These included: Staphylococcus spp. (*S. equorum*, *S. haemolyticus*, *S. lentus*, *S. napalensis*, *S. simulans*, *S. xylosus)*, *Enterococcus faecium*, *Macrococcus canis*, *Micrococcus luteus*, *Bacillus pumilus*, *Aerococcus viridans*, *Klebsiella oxytoca*, *Leclercia adecarboxylata*, *Acinetobacter spp. (A. lwofii*, *A. radioresistens)*, *Enterobacter cloacae*, *Psychrobacter pasteurii*, *Pantoea* spp., *Glutamicibacter* spp.

Bacteria that were isolated only from the dams included: *Streptococcus canis* (V), *Enterococcus canis* (R), *Lactobacillus* spp. (R), *Bacillus cereus* (V), *Kurthia* spp. (V), haemolytic *E. coli* (R, V), *Klebsiella variicola* (R), *Enterococcus hirae* (R) and *Citrobacter* spp. *(R).*

*Enterococcus faecalis* was the most frequently isolated bacterium from the puppies’ meconium and the genus *Enterococcus* was generally one of the more represented in the dams’ rectum and vagina (88.9% and 55.6%, respectively).

*Clostridium perfringens* was isolated only in two puppies of the same ACD litter, while five dams (55.6%) harboured the bacterium (three dams in the rectum, one in the vagina and the fifth both in rectum and vagina).

As for the environmental samples, only few bacterial species were found (*Enterococcus faecium*, *Bacillus cereus* and *Psychrobacter* spp., all with low bacterial load). From ambient air only *Psychrobacter* spp. was isolated.

### Next-generation sequencing (NGS)

Bacterial population was also investigated by genomic approach.

**Fig. 1:**
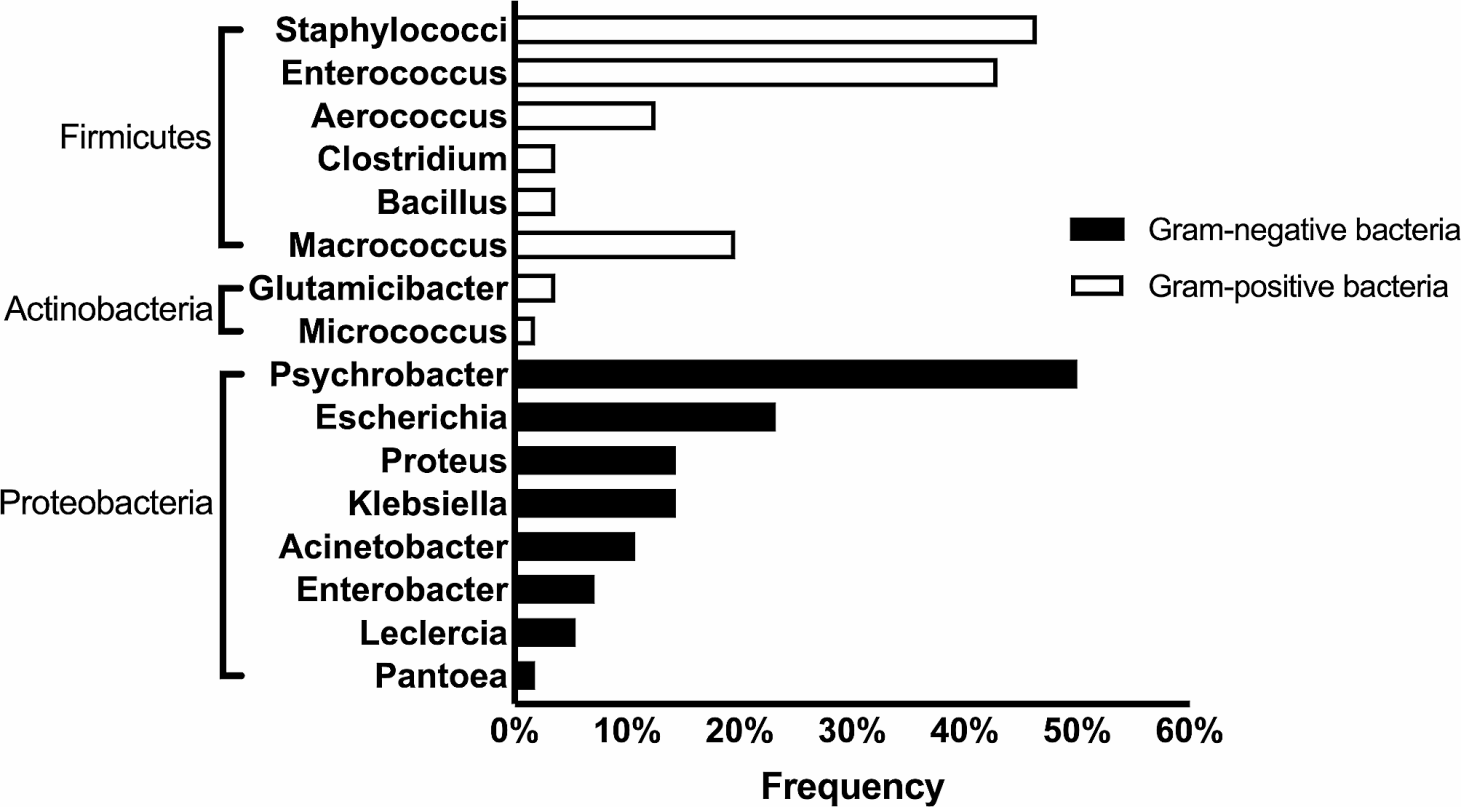
Frequency of bacterial genera (%) isolated from meconium samples. Positive samples (*N* = 56) presented coagulase positive and negative Staphylococci (46.4%), *Enterococcus* (42.9%), *Macrococcus* (19.6%), *Aerococcus* (12.5%), *Clostridium* (3.6%), *Bacillus* (3.6%), *Micrococcus* (1.8%), *Psychrobacter* (50.0%), *Escherichia* (23.2%), *Proteus* (14.3%), *Klebsiella* (14.3%), *Acinetobacter* (10.7%), *Enterobacter* (7.1%), *Leclercia* (5.4%), *Glutamicibacter* (3.6%), and *Pantoea* (1.8%)

Taxonomic composition showed that meconium samples primarily consisted of bacteria that are members of the phyla Proteobacteria (median percent of sequences = 33), Bacteroidetes (median percent of sequences = 16), Firmicutes (median percent of sequences = 13), Actinobacteria (median percent of sequences = 6) and Fusobacteria (median percent of sequences = 6). Figure 1 shows the phylum to which the genera of bacteria isolated in culture belong.

Considering the predominant phylum Proteobacteria, 36.67% of the reads were attributed to the class Alphaproteobacteria, mainly represented by the family Phyllobacteriaceae (61.44%), 33.72% of the reads were assigned to the class Gammaproteobacteria and 25.79% of the reads to the Betaproteobacteria, among which the order Burkholderiales was the most abundant (81.44%).

As for the phylum Bacteroidetes, a little less than a quarter (21.55%) of the reads was assigned to the family Chitinophagaceae, followed by the family Flavobacteriaceae (7.94%).

Within the phylum Firmicutes, Bacilli (46.40%) and Clostridia (43.02%) accounted for most of the reads. Bacillales was the most abundant order (37.86%) in the class Bacilli, whereas Lachnospiraceae (37.75%), Clostridiaceae (31.92%), Peptostreptococcaceae (23.28%) were the most represented families in the class Clostridia.

Within the phylum Actinobacteria, most of the reads were attributed to the order Actinomycetales (94.72%), and particularly to the families Micrococcaceae (10.7%) and Intrasporangiaceae (10.4%).

Finally, Fusobacteriaceae was the most represented family (99.3% of the reads) belonging to the phylum Fusobacteria.

In maternal rectal samples the predominant phyla were Fusobacteria and, with lower frequency than in meconium, Actinobacteria and Proteobacteria. Vaginal samples were less homogeneous, displaying a more variable frequency of the cited phyla.

**Fig. 2:**
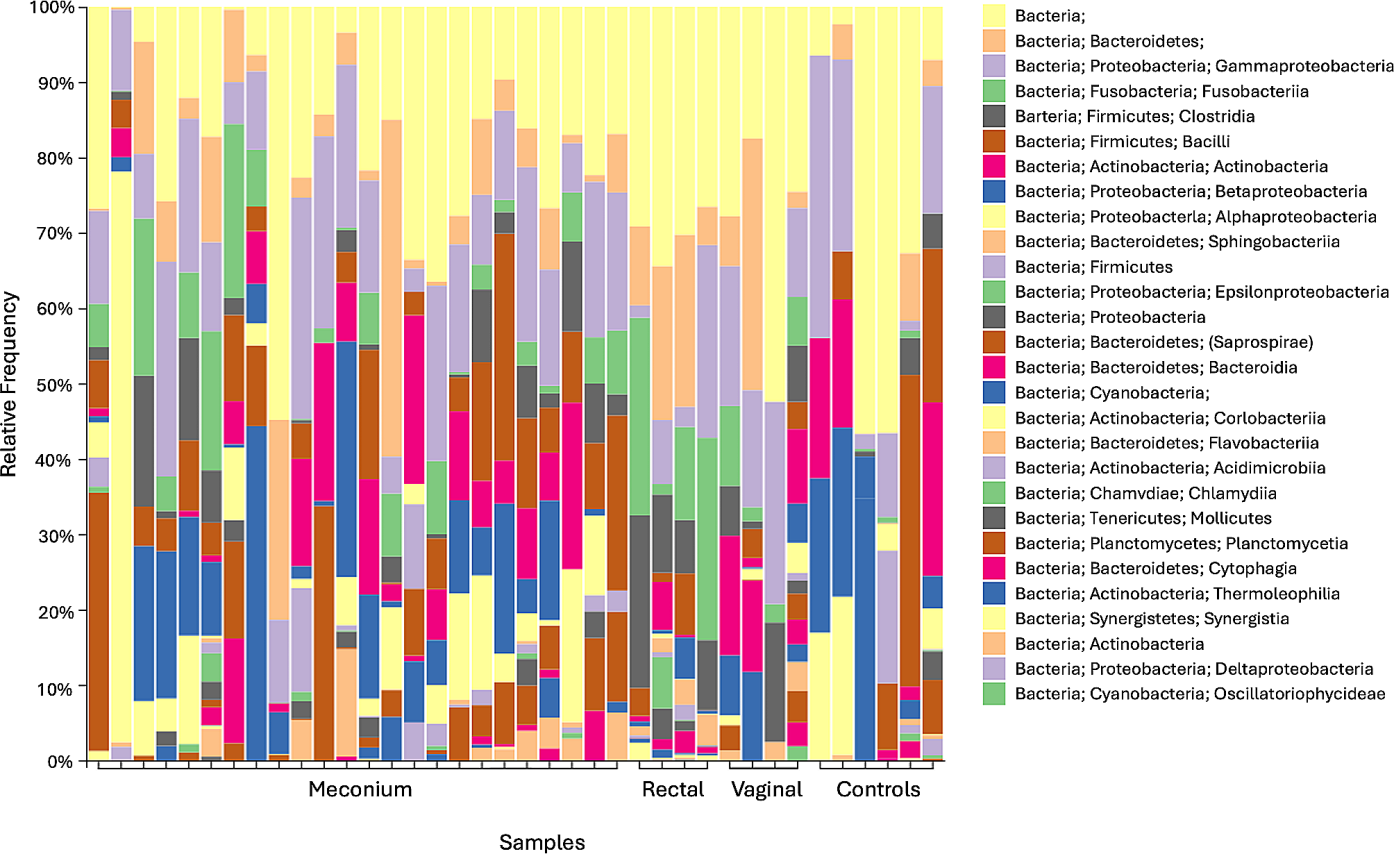
Relative abundance of bacterial sequences detected by 16S rRNA sequencing in samples from dams and their puppies. Samples are grouped by tissue type.

The results at phylum level are shown in Fig. 2.

**Fig. 3:**
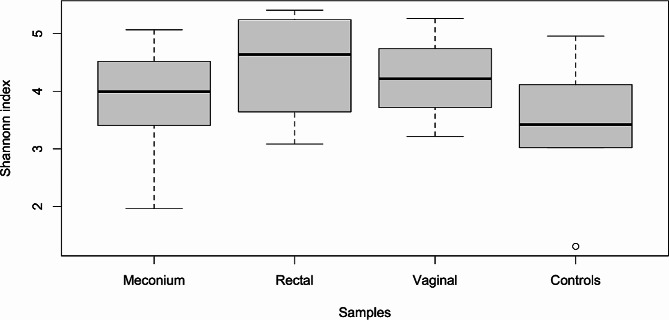
Alpha diversity among different sample types (puppies’ meconium, dams’ rectum and vagina, environmental controls). No significant differences were detected.

Alpha diversity revealed a uniform behaviour among tissues, meaning that meconium samples had similar diversity as mother samples. Shannon index was similar in samples from the same tissue (Fig. 3) and if samples from the same family were compared (Kruskal Wallis rank sum test *p* = 0.0989 and *p* = 0.3326 respectively).

**Fig. 4:**
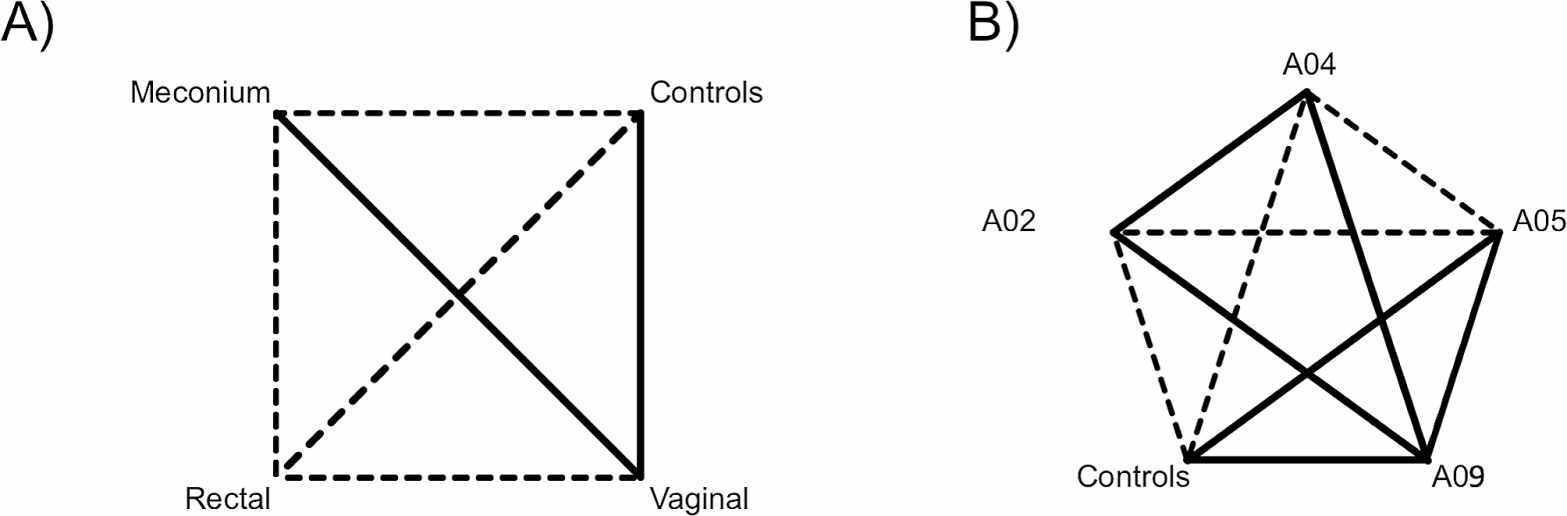
Schematic representations of Beta diversity among (A) tissues and (B) families. Solid lines represent not significant differences (PERMANOVA q > 0.05), dashed lines represent significant differences (PERMANOVA q < 0.05)

Beta diversity indices, calculated by Qiime2, indicated a particular behaviour among tissues: all the tissues were statistically different from each other (PERMANOVA q < 0.05), excluding meconium and vaginal samples (PERMANOVA q = 0.323) and vaginal and control samples (PERMANOVA q = 0.1932) (Fig. 4A).

Moreover, beta diversity analysis showed differences between some families, presenting a quite different behaviour (Fig. 4B). The largest part of the comparisons was significant: this result highlighted a different microbiome among different families and between canine and the environmental samples.

**Fig. 5:**
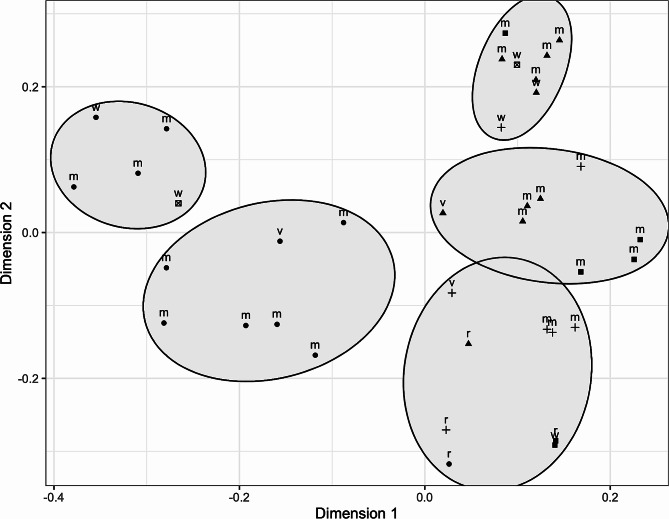
Multidimensional scaling representing Jaccard distances among NGS sequenced samples. Clusters were identified by k-means algorithm with K = 5. The shape of each sample indicates the family membership (circle: A02, triangle: A04, square: A05, cross: A09). Labels close to each sample indicate the tissue origin (m: meconium, r: rectal, v: vaginal, w: white/environment)

To depict all the information together, multidimensional scaling representation based on the Jaccard distance was used to show the relationship among all samples. In Fig. 5 the spatial distribution of samples is shown. Association indexes revealed a significant correlation between the clusters identified by k means algorithm and both the family membership (Pearson’s Chi-squared test *p* < 0.01) and the tissue origin (meconium, rectum, vagina, or environment, Pearson’s Chi-squared test *p* < 0.01).

## Discussion

In the present study, the seeding of the meconium microbiota of canine newborns was investigated on a population of 60 puppies belonging to nine litters. Both culture dependent and molecular methods were applied, although the sequencing of the 16 S rRNA bacterial gene was limited to 24 puppies belonging to four litters. Our observations confirm the immediate colonization of the newborn gut, with a strong and characterizing maternal contribution.

The early development of the gut microbiota is believed to play a key role in perinatal life and in the future health of the individual, because the interactions between the newborn host and the commensal/symbiotic microorganisms shape the immunologic system and exert ‘priming’ effects on metabolic tracts [1]. The association between meconium microbiota composition and puppy health requires the description of the initial microbial colonization and the later baseline intestinal microbiota profile.

‘Priority effects’ (i.e., the influence of microorganisms altering an ecological niche on further possible colonization by other microbial immigrants) [11] have been hypothesized in humans, suggesting that the order of arrival of bacterial strains immediately after birth may influence the subsequent composition of the gut microbiota [12–15]. No previous studies in dogs investigated the early moment in which the puppy has just exit the vaginal canal, nor molecular techniques have ever been applied to this scope. Only one study assessed the bacterial flora of the meconium of newborn puppies by culture; however, samples were not collected at birth, but after colostrum intake, adding time to colonization, bacterial multiplication, and further possible sources of bacteria [7]. The sterility of meconium during foetal life has been challenged in humans and animals [6, 9, 16–24]. However, most of the studies that detected bacteria populations in human foetuses from healthy pregnancies have been shown to have strong pitfalls [4]. Time and mode of collection are critical factors in investigating meconium microbiome because bacteria can be the result of microbial exposure in the birth canal and subsequent multiplication when the meconium is collected long after delivery [5, 25]. When term foetal meconium was collected at non-laboured, elective caesarean deliveries, adopting strict measures to reduce risk of contamination, the comparison with appropriate negative controls showed no microbiome before birth [9]. Analogous preliminary observations were done, both with traditional culture and genomic analyses, in feline and canine foetuses at term, extracted through elective caesarean sections: only microorganisms that can be ascribed to contaminants deriving from the skin of the mother or from the environment were found, or bacteria DNA of unproven viability [8]. Other studies that had reported the existence of a foetal meconium microbiome had included naturally delivered infants [24] or had analysed samples of meconium collected from diapers within 24 hours from birth [17, 20]. Detecting the exact timing of seeding is essential to deem the source of microbial populations in neonates. If the uterine environment is free from living bacteria, bacterial colonization begins during the birth process and the newborn gut is exposed to maternal flora and environmental sources.

Our results show that immediately after birth, the distal portion of the newborn puppy intestine is colonized by bacteria of maternal and environmental origin. Differently from humans, the birth canal may not be the major source of the neonatal flora in dogs, because the amniotic membrane is generally intact when puppies are born and the bitch tears it with her teeth [26, 27]. All our observations were done on puppies that had not contact with the dam’s mouth, allowing to assess the maternal (vaginal or rectal) and environmental contributions to the initial seeding. Previous observations on canine neonates showed that the entire gastrointestinal tract is colonized on the first day of life, and aerobic and anaerobic bacteria were isolated in the distal portion of the colon [28]. Sequencing of human meconium collected within 16 h of delivery showed a high level of human DNA and low levels of microbial DNA [2]. In our investigation, culture revealed that bacteria of the genera *Staphylococcus*, *Enterococcus*, *Escherichia* and *Proteus* are commonly isolated; lactic bacteria (*Micrococcu*s and *Macrococcus*) were isolated less frequently. These seem to mirror the maternal component, although ubiquitous/environmental bacteria (*Acinetobacter*, *Psychrobacter*) are also common. Thence, the prevailing bacteria were aerobic, with a sporadic presence of facultative anaerobes like *Enterobacter cloacae* and *Bacillus pumilus* and anaerobic bacteria like *Clostridium perfringens*. In humans, Bittinger et al. [2], observed an early shift in meconium composition, from facultative anaerobes (e.g., Bacilli and Enterobacteriaceae) to strict anaerobes (e.g., Bacteroides, Clostridium) twenty-five hours after birth. Accordingly, in dogs aerotolerant bacteria dominated in the first day of life but during the following days the anaerobic bacteria increased in absolute and relative numbers [28]. Our study takes a picture of an earlier time, immediately after birth, which can be coherent with the prevalence of aerobic bacteria.

The role of the bacteria of the genus *Enterococcus*, one of the more represented in puppies’ meconium and in dams’ rectum and vagina, deserves further attention. Its prevalence in healthy newborns might suggest that bacteria belonging to this genus could be markers of a normal and balanced early gut flora.

We did not record whether puppies were born within intact amniotic sacs or not: in the first case, the initial colonization is likely to have stronger environmental components, also due to the assistance from the veterinarian, though wearing sterile gloves. When the amniotic sac tears in the passage through the birth canal, the components from maternal vaginal and rectal bacterial communities can rapidly colonize the neonate. Only few species and a low bacterial load were found in environmental samples, reflecting the excellent hygienic conditions in which the deliveries occurred.

Even though next-generation molecular techniques were limited to four dam-litter units, due to costs and materials availability, our preliminary observations confirm the early colonization of the newborn gut.

Results for beta-diversity (i.e., in-between samples diversity) suggested a larger contribution of the maternal vaginal flora to the initial seeding of the canine neonate. Zakosek Pipan et al. [7] drew the same conclusion, although puppies’ meconium was sampled after the first colostrum intake and only culture-dependent techniques were used. In contrast, some studies in humans indicated that the infant pioneer microbiome resembles the faecal populations of the mother, due to contact during birth [29, 30], albeit the colonization by vaginal microbial populations has also been reported [31]. Nevertheless, whatever the initial source, this is rapidly obscured by one to three days postpartum [31]. The infant gut microbiome at birth shows low bacterial load, that increases in the first days of life [32] along with an increase in alpha and a decrease in beta diversities [33, 34]. Also Guard et al. [10] revealed evident shifts in the gut microbiota of puppies from 2 to 56 days after birth and showed modifications that included increased microbial diversity and species richness.

Interestingly, in the present study alpha-diversities were not different based on the sample type. The limited number of maternal and environmental samples compared to the far larger number of meconium ones, might have influenced this result.

As for the composition, meconium samples primarily consisted of bacteria belonging to the phyla Proteobacteria, Firmicutes, and Actinobacteria, confirming the results obtained through traditional culture techniques, by which bacterial species belonging to the same phyla were isolated. Not surprisingly, sequencing revealed the presence of further bacteria, due to its higher sensitivity [35]. For instance, no bacteria belonging to the phyla Bacteroidetes and Fusobacteria were isolated in culture. This might be associated with the selection operated by culture media or with their non-viability. Bacterial viability can only be verified by culture.

In the work of Guard et al. [10], Firmicutes dominated at day two, followed by Proteobacteria and Fusobacteria, with a median percent of sequences of 64.3, 12.5 and 4.5, respectively. Actinobacteria represented less than 1% of all sequences, similarly to Bacteroidetes. Our observations at birth show different proportions: Proteobacteria resulted as the most abundant phylum (*N* = 33), followed by Bacteroidetes (*N* = 16) and Firmicutes (*N* = 13). Actinobacteria and Fusobacteria were the least present, however each-one with a median percent of sequences of 6. We can suppose a very quick modification, although it cannot be excluded that the different protocols, in terms of extraction kits, sequenced regions and sequencing platform, might have affected the results.

A key finding of the present study is the strong similarity in microbiota among family members (i.e., dam-litter units), more than between different families (i.e., dam-litter units): since the dams shared the same environment and the same diet, this means that the imprint of the meconium microbiota of a litter is given by the individual maternal bacterial flora. These results confirm the strong role that the dam has in shaping the gut microbiota of her puppies, role that was recently recognized by similar result obtained with culture dependent methods [36]. The dam appears to vertically transfer components of her microbiota and to give a signature to that of her litter: this finding could have clinical implications and is worth investigating in further studies and in subsequent puppies’ ages and adulthood. Whether the dynamics of the microbial population throughout the paediatric period are associated with health conditions is a main field for future research.

Further investigations are also necessary to assess the effects of different factors, such as delivery mode or intrapartum antimicrobial prophylaxis, on the microbiota of canine neonates. Few studies were carried out on these topics and using only culture methods [7, 37].

Recent works failed to support in-utero bacterial colonization for the human [4, 9] and canine gut [8] but it may still be worth assessing whether a selective passage from dam to foetus, limited to some bacteria genera, could be hypothesized in dogs, as suggested by studies in pregnant rats that showed the vertical transmission of orally administered bifidobacterial [38]. Whether these microorganisms provide relevant immunological stimulation and if this is exerted by the viable bacteria or by their components is still to be elucidated.

## Conclusion

In conclusion, this study confirmed that the meconium of vaginally delivered puppies has its own microbiota immediately after the passage throughout the vaginal canal, and that such microbiota is shaped by the dam, even before any maternal care is provided to the puppies. Future research could investigate the role that oral maternal microbiota has on the puppies’ microbiota composition. Since the dam seeds the initial bacterial populations of her litter, future research could also be directed to assess how modulating maternal microbiota affects that of the offspring, aiming at improving puppies’ health.

## Methods

### Animals and sampling

The study was approved by the Ethical Committee of the Department of Veterinary Sciences of the University of Turin (Approval number 2200, 24/09/2019) and was performed in accordance with the EU Directive 86/609/CEE and with the guidelines of the Italian Ministry of Health for the care and use of animals (D.L. 4 March 2014 n. 26 and D.L. 27 January 1992 n. 116). Previous informed consent was obtained from the dog breeders.

Nine litters of three Appenzeller Cattle Dog (ACD) and six Lagotto Romagnolo (LR) bitches, housed in the same breeding kennel, were included in the study. They represented all the naturally born litters, delivered after uneventful pregnancies, during the observation period. No antimicrobials or other drugs or supplements were administered during pregnancy and only the usual deworming treatment was done ten days before the expected parturition date (Milbactor^®^ Krka, d.d. Novo Mesto, Slovenia). The bitches differed for age and parity and delivered 54 alive and 6 stillborn puppies (Table 1).

They were kept in indoor spaces with access to outdoor yards and fed with the same commercial food (MONGE Natural Superpremium Medium Adult Rich in Chicken^®^, Monasterolo di Savigliano, Cuneo, Italy), shifting towards a dry balanced diet for growing medium size dogs (MONGE Medium Puppy & Junior Rich in Chicken^®^, Monasterolo di Savigliano, Cuneo, Italy) in the last two weeks of pregnancy. Food quantity was calculated based on FEDIAF Guidelines 2019 [39].

Concurrently with the diet change, the bitches were transferred to the nursery area, where smaller kennels with tiled floor and walls are present, and cleaning with chlorine-based products is done twice daily. Specific outdoor yards are dedicated to this kennel area.

Rectal samples were taken from each puppy immediately at birth, after opening the amniotic sac, when intact. Respiration was stimulated by rubbing the puppy with a clean towel when deemed necessary, and, analogously, the umbilical cord was clamped before presenting the puppy to the dam. Samples were collected by a single operator wearing gloves, and using sterile mini nylon flocked swabs (ESwab, 484CE, Copan Italia Spa, Brescia, Italy); stillborn puppies were sampled as well.

Swabs were collected from the dams’ vagina and rectum, by the same operator, at the end of parturition. Sterile nylon flocked swabs (ESwab, 480CE, Copan Italia Spa, Brescia, Italy) were introduced in the rectum for faecal sampling and, through a sterile guide, in the vagina. A polypropylene tube (the plastic cover of the Heinz Herenz Dry Sample Collection Swab, Fisher Scientific Italia, Segrate, Milano, Italy) was inserted into the vagina as a sterile guide, after careful opening of the vulvar labia.

Environmental samples were collected before parturition, rubbing the tip of the swabs (ESwab, 480CE, Copan Italia Spa, Brescia, Italy) on the floor of the nursery and in the parturition box, before allowing the dam in. Another swab was exposed to ambient air to collect a sample as control.

Samples were always collected in duplicate, one for culture and another one for metagenomic analysis. Swabs intended for culture were placed into modified liquid Amies medium (ESwab Copan Italia Spa, Brescia, Italy) and immediately sent to the laboratory of the Istituto Zooprofilattico Sperimentale delle Venezie (Legnaro, Italy), in refrigerated boxes, and processed within 48 h. Swabs intended for metagenomic analysis were immediately stored at -20 °C and subsequently transferred at -80 °C and processed together at the Department of Veterinary Sciences of the University of Turin (Grugliasco, Italy).

### Bacterial isolation and identification

Bacterial isolation was performed according to standard laboratory culture techniques. Briefly, each swab was diluted in 1 ml of nutrient broth (HIB, Heart Infusion Broth, Biolife, Milan, Italy). Ten and 100 µl of bacterial suspension were respectively inoculated into solid media and broths, as described below.

Search for aerobic microorganisms was conducted using nutrient medium (BA, Blood Agar Base n° 2, Biolife, Milan, Italy) with 5% defibrinated sheep blood (Allevamento Blood, Teramo, Italy), nutrient broth (HIB), and selective Enterobacteriaceae medium (McConkey agar, Biolife, Milan, Italy), Bile-Esculin Azide Agar (BEA, Biolife, Milan, Italy). Cultures were inoculated and incubated at 37 °C ± 1 °C in aerobic conditions.

Search for anaerobic microorganisms was conducted using nutrient medium (BA), selective medium for *Clostridium perfringens* (TSC Agar Base, Biolife, Milan, Italy) and Fluid Thioglycollate medium (THG, Biolife, Milan, Italy). Cultures were inoculated and incubated at 37 °C ± 1 °C under anaerobic conditions.

Culture media were checked at 24–48 h depending on the aerobic or anaerobic conditions; in case of absence of bacterial growth on the plates or turbidity in the nutrient broths, the plates were respectively re-incubated for further 24–48 h, under the same conditions. Broth seeding was performed as previously described [36]. All the microbial colonies grown on the first isolation plates were counted. Based on the number of colony forming units (CFUs), growth was classified as Low (1–10 CFU/10 µL), Moderate (11–30 CFU), or High (≥ 31 CFU).

Bacterial genus identification was phenotypically performed by macroscopic observation of colonies, Gram stain reaction, cellular morphology observation, growth on selective medium, catalase, oxidase, and mobility test; on catalase positive Gram-positive cocci, coagulase tube test was also performed.

Species identification was performed by MALDI-TOF MS: Microflex LT instrument (MALDI Biotyper, Bruker Daltonics) equipped with FlexControl software (version 3.3, Bruker Daltonics).

### Next-generation sequencing (NGS)

The samples collected from four dam-litter units, two ACD (A02, A05, Table 1) and two LR (A04, A09, Table 1), including 24 puppies, were subjected to NGS analysis. Two hundred microliters of sterile ice-cold Phosphate Buffered Saline (PBS) were added to each frozen rectal swab and mixed thoroughly by pulse-vortexing. Bacterial genomic DNA from the samples (*n* = 32) and corresponding negative blank controls (*n* = 6) were extracted from rehydrated swabs and meconium slurry using the RNeasy Power Microbiome KIT (Qiagen, Hilden, Germany) following the manufacturer’s instructions. One microliter of RNaseA (Thermo Fisher Scientific, Waltham, MA, USA) was added to digest RNA, with an incubation of 1 h at 37 °C. DNA was quantified with fluorimetric method Qubit High Sensitive dsDNA kit (Life Technologies, Carlsbad, CA, USA) and standardized at 5 ng/µl.

The 16S rRNA gene was amplified following the Illumina 16S Metagenomic Sequencing Library Preparation Protocol (Illumina Inc. San Diego, CA, USA), with minor modifications. Briefly, the V3-V4 region of the 16S gene was amplified with unique barcoded Polymerase Chain Reaction (PCR) primers containing the Illumina adapter overhang nucleotide sequences: 16S Forward Primer (5’- TCGTCGGCAGCGTCAGATGTGTATAAGAGACAGCCTACGGGNGGCWGCAG) and16S Reverse Primer (5’’-GTCTCGTGGGCTCGGAGATGTGTATAAGAGACAGGACTACHVGGGTATCTAATCC). Polymerase Chain Reaction amplicons were cleaned up and size selected using NucleoMag^®^ NGS Clean-up and Size Select (Macherey-Nagel, Allentown, PA, USA). The resulting products were tagged by using the Nextera XT Index Kit (Illumina Inc., San Diego, CA, USA). After the second purification step, amplicon products were quantified using Qubit High Sensitive dsDNA kit (Life Technologies, Carlsbad, CA, USA). Purified and normalized libraries were then pooled and diluted to a 4 nM concentration. The pooled library was then denatured with 0.2 N NaOH, diluted to 10 pM, and combined with 20% (vol/vol) denatured 10 pM PhiX and sequenced with the MiSeq Illumina platform (Illumina Inc., San Diego, CA, USA) with V3-600 cycles chemistry.

### Analysis of data

Sequencing results were analysed using Trimmomatic and Qiime 2-2019.10 standard pipelines and Green Gene (13-8-99-515-806) database for the taxonomical identification. Alpha (Shannon index) and Beta (Jaccard distance matrix) diversities were calculated (among tissues and among families). Jaccard distance matrix was used to assess the bacterial population structure. Based on the obtained distance matrix by Qiime pipeline, cluster analysis with k groups was conducted using R statistical software: k parameter was equal to 5 for both the grouping methods (tissues origin or family membership, including blank samples); the association between cluster membership and biological features was evaluated by the Χ^2^ association index.

### Electronic supplementary material

Below is the link to the electronic supplementary material.

Additional file 1: Percentage of bacterial species identified in the samples, divided for dam and sample origin (Description of data: M: meconium samples; V: vaginal samples from the dam; R: rectal samples from the dam. Bacteria genera are written in bold in the first column. Meconium samples: the percentages referred to bacterial genera are calculated based on the ratio of positive puppies to litter size. The same calculation is done for the bacterial species: when not all the species belonging to a genus were identified, the sum of the percentages of the identified species is lower than the percentage of the corresponding genus. Vaginal and rectal samples: X = positive sample. ^1^Animals used in NGS analyses)

## Data Availability

The datasets used and/or analysed during the study are available from the corresponding author on reasonable request.

## Abbreviations

ACD: Appenzeller Cattle Dog
CFU: Colony-forming unit
LR: Lagotto Romagnolo
MALDI-TOF: MS matrix-assisted laser desorption/ionization coupled to time-of-flight mass spectrometry
NGS: Next-generation sequencing
