# Supplementary material for: Meconium microbiota in naturally delivered canine puppies
Source: BMC Vet Res. 2024 Aug 12;20:363. doi: 10.1186/s12917-024-04225-2 (PMC11318152; doi:10.1186/s12917-024-04225-2)
Supplement: Supplementary file 1 — Additional file 1: Percentage of bacterial species identified in the samples, divided for dam and sample origin (Description of data: M: meconium samples; V: vaginal samples from the dam; R: rectal samples from the dam. Bacteria genera are written in bold in the first column. Meconium samples: the percentages referred to bacterial genera are calculated based on the ratio of positive puppies to litter size. The same calculation is done for the bacterial species: when not all the species belonging to a genus were identified, the sum of the percentages of the identified species is lower than the percentage of the corresponding genus. Vaginal and rectal samples: X = positive sample. 1Animals used in NGS analyses) [file 12917_2024_4225_MOESM1_ESM.docx]

**Supplementary material**. Percentage of bacterial species identified in the samples, divided for dam and sample origin.

|  | A01  N=8 | | | A02^1^  N=9 | | | A05^1^  N=4 | | | A04^1^  N=9 | | | A07  N=8 | | | A08  N=4 | | | A09^1^  N=4 | | | A03  N=6 | | | A06  N=8 | | |
| --- | --- | --- | --- | --- | --- | --- | --- | --- | --- | --- | --- | --- | --- | --- | --- | --- | --- | --- | --- | --- | --- | --- | --- | --- | --- | --- | --- |
|  | **M** | **V** | **R** | **M** | **V** | **R** | **M** | **V** | **R** | **M** | **V** | **R** | **M** | **V** | **R** | **M** | **V** | **R** | **M** | **V** | **R** | **M** | **V** | **R** | **M** | **V** | **R** |
| ***Staphylococcus*** | 62.5 | X |  | 22.2 | X |  | 100 | X |  | 66.7 | X | X | 50.0 | X |  | 0 |  | X | 0 |  |  | 50.0 | X |  | 25.0 |  |  |
| *S. equorum* | 0 |  |  | 0 |  |  | 0 |  |  | 11.1 |  |  | 0 |  |  | 0 |  |  | 0 |  |  | 0 |  |  | 0 |  |  |
| *S. haemolyticus* | 25.0 |  |  | 0 |  |  | 0 |  |  | 0 |  |  | 0 |  |  | 0 |  |  | 0 |  |  | 0 |  |  | 0 |  |  |
| *S. lentus* | 0 |  |  | 0 |  |  | 0 |  |  | 0 |  |  | 0 |  |  | 0 |  |  | 0 |  |  | 16.7 |  |  | 0 |  |  |
| *S. napalensis* | 0 |  |  | 11.1 |  |  | 0 |  |  | 0 |  |  | 0 |  |  | 0 |  |  | 0 |  |  | 0 |  |  | 0 |  |  |
| *S. saprophyticus* | 12.5 |  |  | 0 | X |  | 25.0 |  |  | 33.3 |  |  | 0 |  |  | 0 |  |  | 0 |  |  | 0 |  |  | 0 |  |  |
| *S. sciuri* | 12.5 |  |  | 0 |  |  | 50.0 | X |  | 22.2 |  |  | 0 |  |  | 0 |  |  | 0 |  |  | 16.7 | X |  | 0 |  |  |
| *S. simulans* | 12.5 |  |  | 0 |  |  | 0 |  |  | 0 |  | X | 0 |  |  | 0 |  |  | 0 |  |  | 0 |  |  | 0 |  |  |
| *S. xylosus* | 0 |  |  | 0 |  |  | 50.0 |  |  | 11.1 |  |  | 25.0 |  |  | 0 |  |  | 0 |  |  | 16.7 |  |  | 25.0 |  |  |
| *S. aureus* | 25.0 | X |  | 0 |  |  | 0 |  |  | 0 |  |  | 0 |  |  | 0 |  |  | 0 |  |  | 0 |  |  | 0 |  |  |
| *S. pseudintermedius* | 0 |  |  | 11.1 |  |  | 0 |  |  | 0 |  |  | 12.5 | X |  | 0 |  |  | 0 |  |  | 0 |  |  | 0 |  |  |
| ***Streptococcus*** | 0 |  |  | 0 |  |  | 0 |  |  | 0 | X |  | 0 | X |  | 0 |  |  | 0 |  |  | 0 |  |  | 0 |  |  |
| *S. canis* | 0 |  |  | 0 |  |  | 0 |  |  | 0 | X |  | 0 | X |  | 0 |  |  | 0 |  |  | 0 |  |  | 0 |  |  |
| ***Enterococcus*** | 0 | X | X | 33.3 |  | X | 25.0 | X | X | 66.7 |  | X | 87.5 | X | X | 25.0 |  | X | 0 |  |  | 33.3 | X |  | 50.0 | X | X |
| *E. canintestini* | 0 | X | X | 0 |  |  | 0 |  |  | 0 |  |  | 0 |  |  | 0 |  |  | 0 |  |  | 0 |  |  | 12,5 |  |  |
| *E. canis* | 0 |  |  | 0 |  |  | 0 |  |  | 0 |  | X | 0 |  |  | 0 |  |  | 0 |  |  | 0 |  |  | 0 |  |  |
| *E. faecalis* | 0 | X |  | 11.1 |  | X | 0 | X | X | 11.1 |  | X | 87.5 | X | X | 25.0 |  | X | 0 |  |  | 33.3 | X |  | 25.0 | X | X |
| *E faecium* | 0 |  |  | 11.1 |  |  | 0 |  |  | 0 |  |  | 0 |  |  | 0 |  |  | 0 |  |  | 0 |  |  | 25.0 |  |  |
| *E. hirae* | 0 |  |  | 0 |  |  | 0 |  |  | 0 |  |  | 0 |  |  | 0 |  |  | 0 |  |  | 0 |  | X | 0 |  |  |
| ***Macrococcus*** | 0 |  |  | 0 |  |  | 0 |  |  | 22.2 |  |  | 37.5 |  |  | 25.0 |  |  | 0 |  |  | 50.0 | X |  | 25.0 |  |  |
| *M. canis* | 0 |  |  | 0 |  |  | 0 |  |  | 11.1 |  |  | 25.0 |  |  | 25.0 |  |  | 0 |  |  | 50.0 |  |  | 25.0 |  |  |
| *M. caseolyticus* | 0 |  |  | 0 |  |  | 0 |  |  | 11.1 |  |  | 0 |  |  | 0 |  |  | 0 |  |  | 0 | X |  | 0 |  |  |
| ***Lactobacillus*** | 0 |  |  | 0 |  |  | 0 |  |  | 0 |  |  | 0 |  |  | 0 |  |  | 0 |  | X | 0 |  |  | 0 |  |  |
| *L. murinus* | 0 |  |  | 0 |  |  | 0 |  |  | 0 |  |  | 0 |  |  | 0 |  |  | 0 |  | X | 0 |  |  | 0 |  |  |
| ***Micrococcus*** | 0 |  |  | 0 |  |  | 0 |  |  | 11.1 |  |  | 0 |  |  | 0 |  |  | 0 |  |  | 0 |  |  | 0 |  |  |
| *M. luteus* | 0 |  |  | 0 |  |  | 0 |  |  | 11.1 |  |  | 0 |  |  | 0 |  |  | 0 |  |  | 0 |  |  | 0 |  |  |
| ***Clostridium*** | 0 |  | X | 22.2 |  |  | 0 |  |  | 0 |  |  | 0 |  | X | 0 |  | X | 0 |  | X | 0 | X | X | 0 | X |  |
| *C. perfringens* | 0 |  |  | 22.2 |  |  | 0 |  |  | 0 |  |  | 0 |  | X | 0 |  | X | 0 |  | X | 0 | X | X | 0 | X |  |
| ***Bacillus*** | 12.5 | X |  | 0 |  |  | 0 |  |  | 0 |  |  | 0 |  |  | 0 |  |  | 0 | X |  | 0 |  |  | 12.5 |  |  |
| *B. cereus* | 0 |  |  | 0 |  |  | 0 |  |  | 0 |  |  | 0 |  |  | 0 |  |  | 0 | X |  | 0 |  |  | 0 |  |  |
| *B. pumilus* | 12.5 |  |  | 0 |  |  | 0 |  |  | 0 |  |  | 0 |  |  | 0 |  |  | 0 |  |  | 0 |  |  | 12.5 |  |  |
| ***Aerococcus*** | 12.5 |  |  | 0 |  |  | 0 |  |  | 33.3 |  |  | 0 |  |  | 75.0 |  |  | 0 |  |  | 0 |  |  | 0 |  |  |
| *A. viridans* | 12.5 |  |  | 0 |  |  | 0 |  |  | 33.3 |  |  | 0 |  |  | 0 |  |  | 0 |  |  | 0 |  |  | 0 |  |  |
| ***Kurthia*** | 0 |  |  | 0 |  |  | 0 |  |  | 0 | X |  | 0 |  |  | 0 |  |  | 0 |  |  | 0 |  |  | 0 |  |  |
| *K. zopfii* | 0 |  |  | 0 |  |  | 0 |  |  | 0 | X |  | 0 |  |  | 0 |  |  | 0 |  |  | 0 |  |  | 0 |  |  |
| ***Escherichia*** | 12.5 | X | X | 66.7 |  | X | 100 | X | X | 0 |  | X | 25.0 | X | X | 0 |  | X | 0 |  | X | 0 | X |  | 0 | X | X |
| *E. coli* | 12.5 | X | X | 66.7 |  |  | 100 | X | X | 0 |  | X | 25.0 | X | X | 0 |  | X | 0 |  | X | 0 |  |  | 0 | X | X |
| *E. coli (hemolytic)* | 0 |  |  | 0 |  | X | 0 |  |  | 0 |  |  | 0 |  |  | 0 |  |  | 0 |  | X | 0 | X |  | 0 |  |  |
| ***Klebsiella*** | 25.0 | X |  | 66.7 |  | X | 0 |  |  | 0 |  |  | 0 |  |  | 0 |  |  | 0 |  |  | 0 |  |  | 0 |  |  |
| *K. oxytoca* | 0 |  |  | 11.1 |  |  | 0 |  |  | 0 |  |  | 0 |  |  | 0 |  |  | 0 |  |  | 0 |  |  | 0 |  |  |
| *K. pneumoniae* | 12.5 | X |  | 44.4 |  |  | 0 |  |  | 0 |  |  | 0 |  |  | 0 |  |  | 0 |  |  | 0 |  |  | 0 |  |  |
| *K. variicola* | 0 |  |  | 0 |  | X | 0 |  |  | 0 |  |  | 0 |  |  | 0 |  |  | 0 |  |  | 0 |  |  | 0 |  |  |
| ***Proteus*** | 50 | X | X | 11.1 |  |  | 0 |  |  | 0 |  | X | 25.0 | X | X | 0 |  |  | 0 |  |  | 0 |  | X | 12.5 | X | X |
| *P. mirabilis* | 50 | X | X | 11.1 |  |  | 0 |  |  | 0 |  | X | 25.0 | X | X | 0 |  |  | 0 |  |  | 0 |  | X | 12.5 | X | X |
| ***Leclercia*** | 37.5 |  |  | 0 |  |  | 0 |  |  | 0 |  |  | 0 |  |  | 0 |  |  | 0 |  |  | 0 |  |  | 0 |  |  |
| *L. adecarboxylata* | 37.5 |  |  | 0 |  |  | 0 |  |  | 0 |  |  | 0 |  |  | 0 |  |  | 0 |  |  | 0 |  |  | 0 |  |  |
| ***Acinetobacter*** | 12.5 |  |  | 11.1 |  |  | 25.0 |  |  | 11.1 |  |  | 0 |  |  | 0 |  |  | 0 |  |  | 0 |  |  | 25.0 |  |  |
| *A. lwofii* | 12.5 |  |  | 0 |  |  | 0 |  |  | 11.1 |  |  | 0 |  |  | 0 |  |  | 0 |  |  | 0 |  |  | 0 |  |  |
| *A. radioresistens* | 0 |  |  | 0 |  |  | 25.0 |  |  | 0 |  |  | 0 |  |  | 0 |  |  | 0 |  |  | 0 |  |  | 25.0 |  |  |
| ***Enterobacter*** | 12.5 |  |  | 22.2 |  |  | 25.0 |  |  | 0 |  |  | 0 |  |  | 0 |  |  | 0 |  |  | 0 |  |  | 0 |  |  |
| *E. cloacae* | 0 |  |  | 22.2 |  |  | 25.0 |  |  | 0 |  |  | 0 |  |  | 0 |  |  | 0 |  |  | 0 |  |  | 0 |  |  |
| ***Citrobacter*** | 0 |  |  | 0 |  | X | 0 |  |  | 0 |  |  | 0 |  |  | 0 |  |  | 0 |  |  | 0 |  | X | 0 |  |  |
| *C. freundii* | 0 |  |  | 0 |  | X | 0 |  |  | 0 |  |  | 0 |  |  | 0 |  |  | 0 |  |  | 0 |  | X | 0 |  |  |
| ***Psychrobacter*** | 0 |  |  | 11.1 |  |  | 50.0 | X |  | 66.7 |  |  | 37.5 |  |  | 75.0 | X |  | 25.0 | X |  | 83.3 | X |  | 87.5 |  | X |
| *P. pasteurii* | 0 |  |  | 11.1 |  |  | 0 |  |  | 0 |  |  | 0 |  |  | 0 |  |  | 0 |  |  | 0 |  |  | 0 |  |  |
| *P. sanguinis* | 0 |  |  | 0 |  |  | 50.0 |  |  | 0 |  |  | 0 |  |  | 0 |  |  | 0 |  |  | 83.3 | X |  | 12.5 |  | X |
| ***Pantoea*** | 0 |  |  | 0 |  |  | 0 |  |  | 0 |  |  | 12.5 |  |  | 0 |  |  | 0 |  |  | 0 |  |  | 0 |  |  |
| ***Glutamicibacter*** | 0 |  |  | 0 |  |  | 0 |  |  | 11.1 |  |  | 0 |  |  | 0 |  |  | 25.0 |  |  | 0 |  |  | 0 |  |  |

M: meconium samples; V: vaginal samples from the dam; R: rectal samples from the dam.

Bacteria genera are written in bold in the first column.

Meconium samples: the percentages referred to bacterial genera are calculated based on the ratio of positive puppies to litter size. The same calculation is done for the bacterial species: when not all the species belonging to a genus were identified, the sum of the percentages of the identified species is lower than the percentage of the corresponding genus.

Vaginal and rectal samples: X = positive sample.

^1^Animals used in NGS analyses.
